# Tacrolimus Increases Nox4 Expression in Human Renal Fibroblasts and Induces Fibrosis-Related Genes by Aberrant TGF-Beta Receptor Signalling

**DOI:** 10.1371/journal.pone.0096377

**Published:** 2014-05-09

**Authors:** Georg Kern, Sabine M. Mair, Susie-Jane Noppert, Paul Jennings, Herbert Schramek, Michael Rudnicki, Gerhard A. Mueller, Gert Mayer, Christian Koppelstaetter

**Affiliations:** 1 Nephrology and Hypertension, Innsbruck Medical University, Innsbruck, Austria; 2 Clinical Immunology and Infectious Diseases, Innsbruck Medical University, Innsbruck, Austria; 3 Physiology, Innsbruck Medical University, Innsbruck, Austria; 4 Rheumatology and Nephrology, University of Goettingen, Goettingen, Germany; Medical University of Graz, Austria

## Abstract

Chronic nephrotoxicity of immunosuppressives is one of the main limiting factors in the long-term outcome of kidney transplants, leading to tissue fibrosis and ultimate organ failure. The cytokine TGF-β is considered a key factor in this process. In the human renal fibroblast cell line TK-173, the macrolide calcineurin inhibitor tacrolimus (FK-506) induced TGF-β-like effects, manifested by increased expression of NAD(P)H-oxidase 4 (Nox4), transgelin, tropomyosin 1, and procollagen α1(V) mRNA after three days. The macrolide mTOR inhibitor rapamycin had similar effects, while cyclosporine A did not induce fibrose-related genes. Concentration dependence curves were sigmoid, where mRNA expression was induced already at low nanomolar levels of tacrolimus, and reached saturation at 100–300 nM. The effects were independent of extracellular TGF-β as confirmed by the use of neutralizing antibodies, and thus most likely caused by aberrant TGF-β receptor signaling, where binding of tacrolimus to the regulatory FKBP12 protein results in a “leaky” TGF-β receptor. The myofibroblast marker α-smooth muscle actin was neither induced by tacrolimus nor by TGF-β1, indicating an incomplete activation of TK-173 fibroblasts under culture conditions. Tacrolimus- and TGF-β1-induced Nox4 protein upregulation was confirmed by Western blotting, and was accompanied by a rise in intracellular H_2_O_2_ concentration. Si-RNA mediated knock-down of Nox4 expression prevented up-regulation of procollagen α1(V) mRNA in tacrolimus-treated cells, but induced procollagen α1(V) expression in control cells. Nox4 knock-down had no significant effect on the other genes tested. TGF-β is a key molecule in fibrosis, and the constant activation of aberrant receptor signaling by tacrolimus might contribute to the long-term development of interstitial kidney fibrosis in immunosuppressed patients. Nox4 levels possibly play a regulatory role in these processes.

## Introduction

The availability of the calcineurin inhibitors (CNIs) cyclosporine (CsA) [1] and tacrolimus (FK-506) [2] has revolutionized transplantation medicine. Currently more than 90% of all patients receiving a renal graft are treated post-transplant with CNIs [3]. However, CNI nephrotoxicity is a major problem, and lesions at least partly attributable to CNI nephrotoxicity can be seen in virtually all histological sections ten years after transplantation [4].

Fibrogenic effects of CNIs have been described in different compartments of the kidney, with main focus on the tubular-interstitial region. Already in 1990, procollagen secretion in murine epithelial cells and fibroblasts exposed to CsA was reported [5]. The knowledge about the role of tacrolimus in fibrosis is more diverse. Similar fibrogenic responses in patients receiving CsA or tacrolimus have been described six and twelve months after renal transplantation [6]. One year after transplantation, control biopsies from tacrolimus-treated patients with stable graft function show a significantly lower TGF-β1 expression compared to CsA-treated ones [7]. However, after a mean period of 22/28 months not only the expression of TGF-β mRNA is higher in the tacrolimus group, but also several markers of fibrogenesis are overexpressed [8]. As a further consequence of activation of TGF-β signaling, interstitial fibrosis is promoted by an increasing production of extracellular matrix (ECM) proteins [9], [10], and induction of epithelial-to-mesenchymal transition (EMT) [11]. In renal fibroblasts a conversion to a myofibroblastic cell type appeared after exposure to TGF-β [12].

The reduced nicotinamide adenine dinucleotide phosphate (NAD(P)H) oxidases produce reactive oxygen species (ROS) by catalyzing electron transport from NAD(P)H to oxygen molecules [13]. NAD(P)H oxidase type 4 (Nox4) has recently been identified as a key molecule in TGF-β-driven fibrosis [14]. Nox4 is most abundant in the kidney [15], and it is a contributor of ROS in renal cells [16]. The physiological role of Nox4 is still not fully elucidated [15], [17]. It is proposed to modulate redox-sensitive signal pathways such as Ras [18], extracellular signal-regulated kinases ERK1 and ERK2 [16], and p38 mitogen-activated protein (MAP) kinase [19]. Nox4 has been reported to be involved in lung myofibroblast activation [14], osteoblast differentiation [20], idiopathic pulmonary fibrosis [21], kidney myofibroblast activation [12], and cardiac differentiation [22]. Attempts to identify specific Nox4 inhibitors have been reported recently [23].

## Subjects and Methods

### Cell culture

The human kidney fibroblast cell line TK-173 [24] was used exclusively in all experiments, except the initial microarray experiments. TK-173 cells were grown to confluence in serum-containing growth medium, and then switched to serum-free medium for experiments. Growth medium was based on our routinely used renal tubule cell medium [25] and was made up from a 1∶1 mixture of DMEM (Gibco 11966-025; Invitrogen, Lofer, Austria) and Ham's F12 (Gibco 21765-029), supplemented with 10% fetal bovine serum (Gibco 10270), Glutamax (100x, Gibco 35050), and Penicillin-Streptomycin (100x, Gibco P4333). In the serum-free medium FCS was replaced by ITS (5 mg/L insuline, 5 mg/L transferrin, and 5 µg/L sodium selenite; Sigma I-1884, Sigma, Vienna, Austria). Cells were grown on uncoated plasticware (Greiner, Kremsmuenster, Austria). Drugs were purchased from Peprotech, Hamburg, Germany (TGF-β1), Tocris Bioscience, Bristol, UK (LY364947, SB431542), and Fujisawa Pharmaceutical/Astellas, Vienna, Austria (tacrolimus). All experiments (except microarrays) were performed at least in triplicates.

For comparative microarray experiments we used the human proximal tubule epithelial cell line RPTEC/TERT1 [26] (purchased from Evercyte, Vienna, Austria). RPTEC/TERT1 medium was based on the serum-free TK173 fibroblast medium, additionally supplemented with 36 ng/ml hydrocortisone (Sigma H0135), and 10 ng/ml epithelial growth factor (Sigma E9644). Confluent cultures were used for experiments.

### Microarray analysis

Cells were exposed to 10 µM of tacrolimus for one day, or three days, resp. Experiments were performed in duplicates. RNA was isolated from cell culture samples by Qiagen RNeasy Mini Kit. In brief, cells were lysed in buffer containing mercaptoethanol, and the lysate was homogenized by passing it through a sterile syringe needle (0,9 mm) several times. RNA was purified over the columns and eluted in RNase-free H_2_O. Cy3-labeled cRNA was generated from total RNA with the LowInput QuickAmp Labeling Kit (Agilent Technologies, 5190-2331). 1,65 ng of cRNA from each sample were hybridized to one-color microarrays (Whole Human Genome 4×44 K Oligo Microarray kit, Agilent Technologies, G4112F) for 17 hours at 65°C. Slides were scanned on an Axon GenePix 4000B scanner (Molecular Dynamics), and the obtained data were analyzed in the Agilent Feature Extraction software (Vers. 9.5.3.1). Data can be accessed online at www.ebi.ac.uk/arrayexpress under accession number E-MTAB-778).

### Real-time quantitative PCR (RT-qPCR)

Total RNA was isolated by phenol extraction, using Tri Reagent (Sigma T9424) with standard laboratory techniques. mRNA was transcribed into cDNA using reverse transcriptase from Moloney Murine Leukaemia Virus (Invitrogen M-MLV-Kit, 28025-021). qRT-PCR was performed on an Applied Biosystems 7500 Fast RT qPCR System. The following pre-made assays by Applied Biosystems were used: NADPH-oxidase 4 (NOX4, Hs01558199_m1), Transgelin 1 (TAGLN, Hs00162558_m1), collagen alpha1-(V) (COL5A1, Hs00609088_m1), tropomyosin 1 (TPM1, Hs00165966_m1), smooth muscle alpha-actin (ACTA2, Hs00909449_m1), and transforming growth factor-β1 (TGFB1, Hs00171257_m1). Ribosomal 18S gene expression was used as internal standard (Applied Biosystems, 4310893E). All results were expressed as linearized values, fold over untreated control.

### Intracellular ROS measurement

Cells grown in 96-well plates were loaded with the cell-permeable H_2_O_2_ indicator 2′,7′-dichlorofluorescin diacetate (DCF-DA) by incubation in serum-free medium containing 3 µM DCF-DA (Sigma 6883; diluted from 10 mM stock in DMSO) for 60 minutes at 37°C/5% CO_2_. After loading, the cells were carefully washed two times with fresh medium within 10 minutes, and fluorescence at 485 nm/535 nm (excitation/emmission) was determined immediately afterward on a 96-well plate reader (Tecan GENios). Fluorescence intensity values were averaged over 12 wells for each condition and normalized to control  = 1.

### TGF-β neutralization by antibodies

TGF-β antibodies (anti-hTGFβ-IgA, Invivogen) were added to the cell cultures at a concentration of 300 ng/ml, to neutralize extracellular TGF- β. In case of the addition of 5 ng/ml TGF-β1 as a positive control, the complete medium containing both TGF-β1 and antibodies was set aside for several minutes to allow complete neutralization before application to the cells.

### Western Blot

Cells from 6-well plates were harvested in ice-cold RIPA-Buffer (Sigma R0278), supplemented with a protease inhibitor mixture (Sigma 8849) and 1 mM sodium orthovanadate (1 mM). For NOX4 measurement, 30 µg of protein per lane were separated on a 10% SDS polyacrylamide gel, and blotted onto a nylon membrane (Hybond P, Amersham Pharmacia). After blocking (5% BSA in Tris-buffered saline with 0.1% Tween; TBS-T), the membrane was incubated with rabbit polyclonal anti-NOX4 antibody (1/1000; Abcam ab60940) and rabbit anti-actin antibody (2 µg/mL; Sigma A2066) as a loading control. For determination of SMAD2 phosphorylation, 20 µg of protein were separated on a 4–12% NuPAGE Bis-Tris gel (Life Technologies), and blotted onto 0,2 µm nitrocellulose membrane (Life Technologies). The membrane was blocked with 5% milk powder in TBS-T and labelled with anti-pSMAD2 (Cell Signaling 3108, 1∶1000) for 2 hours. Enhanced chemiluminescence (ECL) signal from HRP-labelled secondary antibody (Dako) was detected with a CCD-based imaging system.

### si-RNA mediated Nox4 knockdown

TK-173 fibroblasts were lipofected with Nox4 siRNA (Dharmacon D-010194-01) utilizing Dharmacon transfection reagent No. 3. 37 µl of transfection reagent and 7 µl of siRNA (20 µM) were diluted separately in each 320 µl of serum-free medium. The two solutions were then mixed carefully, and the mixture was left standing for 20 minutes at 37°C. In parallel, a transfection solution with non-target control RNA was prepared the same way. Cells were washed with serum-free medium, and 100 µl of the solution were pipetted on each single well of 12-well plates with confluent TK-173 cells. Incubation with drugs was started the next day.

### Statistics

**Figure 1:**
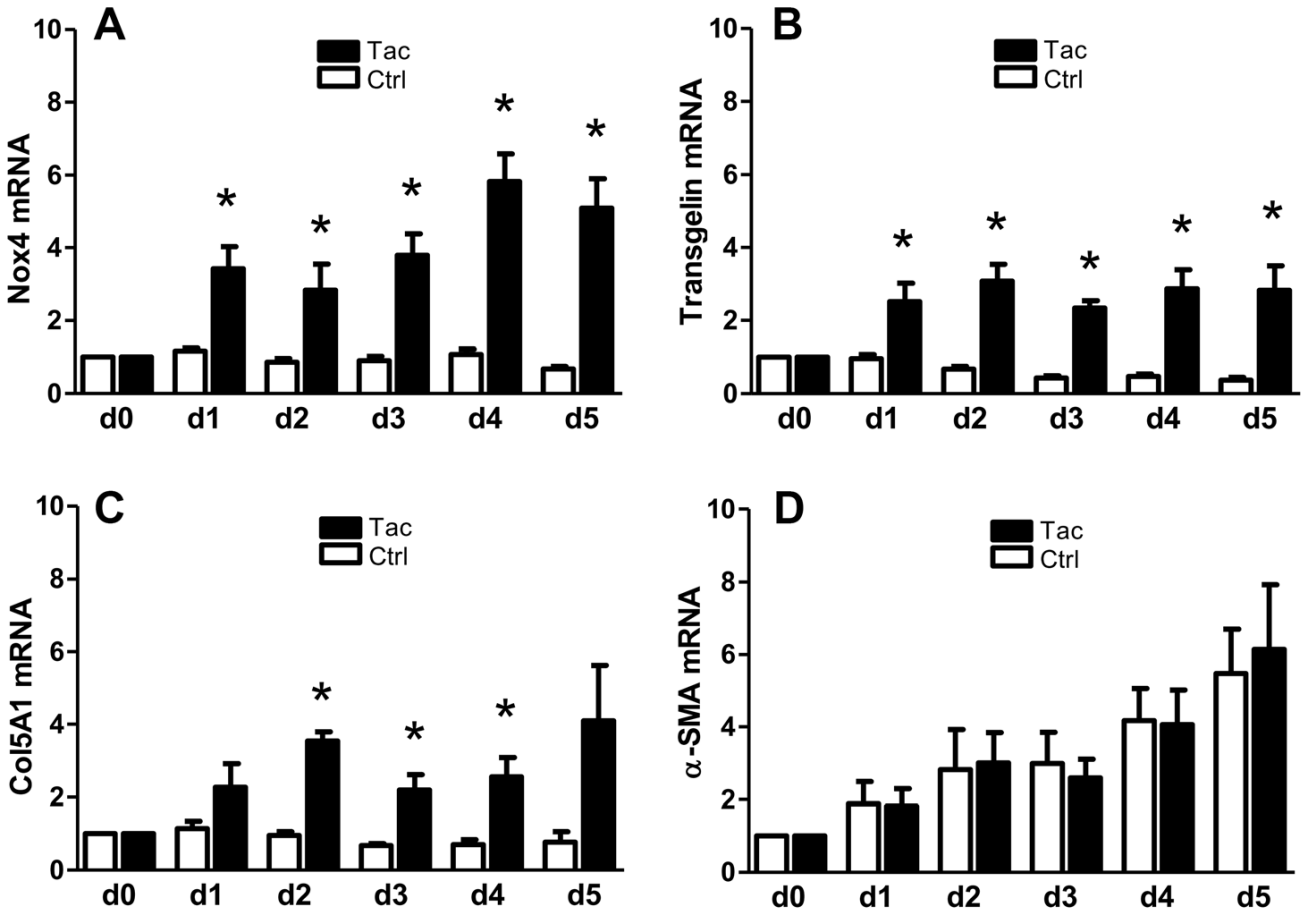
Time course of mRNA expression in TK-173 cells cells treated with 100 nM tacrolimus. Expression of mRNA for NAD(P)H-oxidase 4 (A), transgelin (B), procollagen α1(V) (C), and alpha-smooth muscle actin (D) in untreated cells (open columns), and cells treated with 100 nM tacrolimus (filled). Cells were switched to serum-free medium on day -1, and were then exposed to 100 nM tacrolimus starting at day 0. Cell samples were collected from day 0 to day 5. All results were normalized to day 0. Cells showed a significant response to tacrolimus after one day (Nox4 and transgelin) or two days (tropomyosin-1), respectively. Alpha-smooth muscle actin mRNA showed a slow up-regulation in both treated and untreated cells, most likely as a reaction to serum deprivation. (*) denotes significant (p≤0,05) difference between tacrolimus-treated and control cells at the same timepoint.

One-way *ANOVA* followed by *Bonferroni's* post-hoc comparisons was performed in all statistical analyses, except for the time course experiments (Fig. 1), which were analysed by unpaired t-test combined with *Levene's* test for homogenity of variances. All tests were performed by PRISM software (GraphPad Software, Inc.).

**Figure 2:**
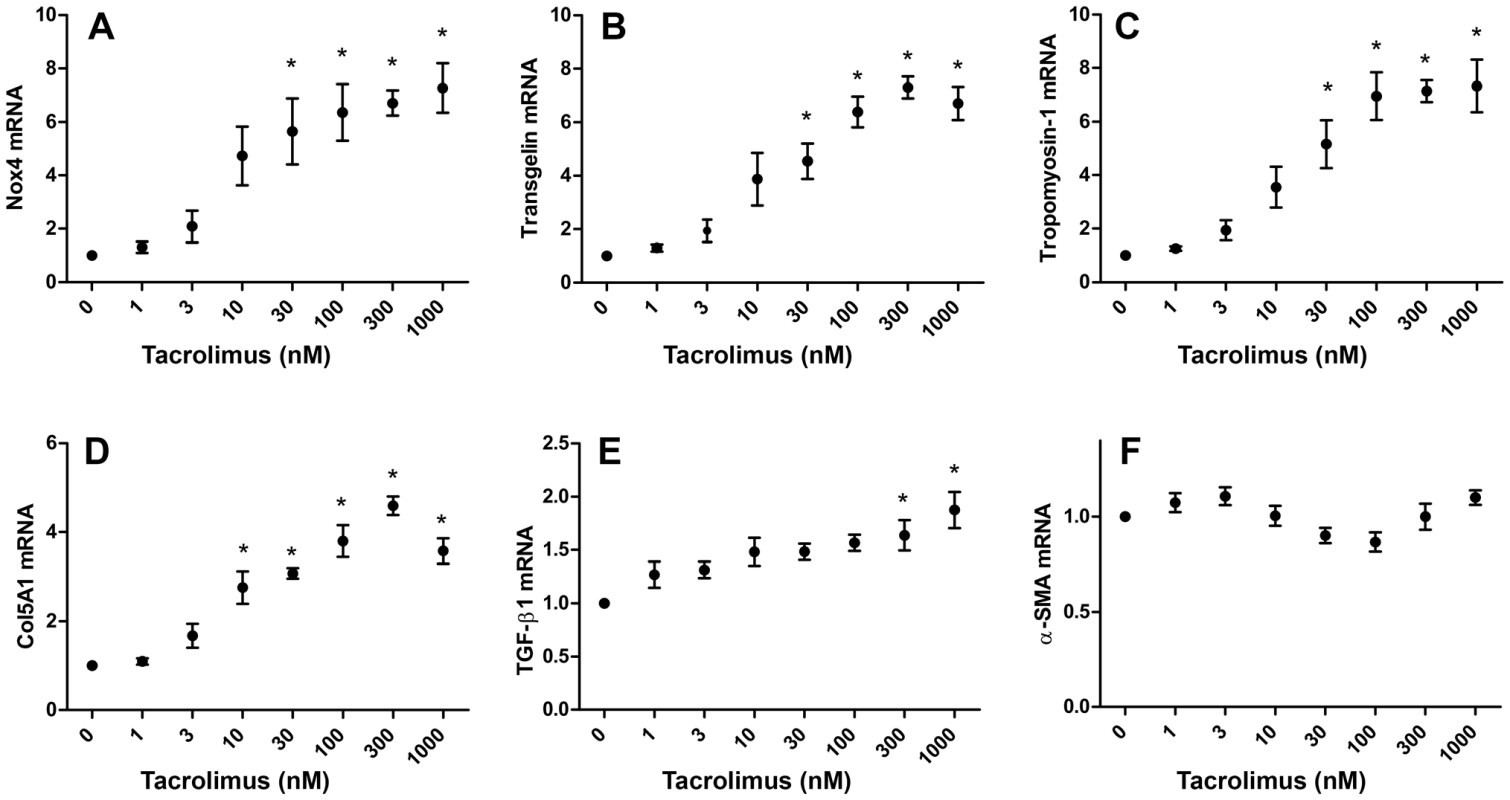
Induction of fibrosis-related genes in TK-173 fibroblasts by nanomolar levels of tacrolimus. Exposure to increasing concentrations of tacrolimus (0–1000 nM) induced mRNA expression in a concentration-dependent manner after three days for Nox4 (A), transgelin (B), tropomyosin-1 (C), procollagen α1(V) (D), and transforming growth factor β-1 (E). a-smooth muscle actin mRNA expression (F) was not affected. RT-qPCR results were standardized to 18S rRNA, linearized, and normalized to the control group (without tacrolimus). (*) indicates significance from untreated cells p≤0,05.

## Results

### Tacrolimus induces up-regulation of fibrosis-related genes in human renal fibroblasts

Human renal fibroblasts (TK-173) and human proximal tubular cells (RPTEC/TERT1) were exposed to 10 µM tacrolimus for one and three days. Statistical analysis of the microarray data identified 211 transcripts that were up-regulated, and 64 that were down-regulated (≥twofold, p≤0.05) in TK-173 fibroblasts after three days. Results after one day were comparable, although with generally lower amplitudes. RPTEC/TERT1 cells showed a much weaker response, with only 25 genes up-regulated and 10 down-regulated. Therefore, we focused further experiments on TK-173 cells. For detailed microarray results see www.ebi.ac.uk/arrayexpress (login data in Methods section).

A subset of fibrosis-related genes was studied in greater detail by real-time quantitative PCR: The cytoskeletal components transgelin (SM22α), tropomyosin 1, alpha (Tpm-1), and alpha-smooth muscle actin (α-SMA, ACTA2); the extracellular matrix component procollagen α1(V); NAD(P)H oxidase 4 (Nox4); and transforming growth factor β-1 (TGF-β1). When treated with tacrolimus concentrations ranging from 1 to 1000 nM for three days, all genes (with the exception of α-SMA) showed a similar concentration-dependent response: the curves had a sigmoid shape, and effects became noticeable already at low nanomolar concentrations of tacrolimus (Fig. 2). From 100–300 nM of tacrolimus on, the effects became saturated, and the RT-qPCR results at high concentrations validated the values from the microarray experiments. The strongest positive reactions were observed on the expression of Nox4 (max. 7,3-fold), tropomyosin 1 (max. 7,3), and transgelin (max. 6,7). α-SMA was not regulated by exposure to tacrolimus (Fig. 2F), neither in RT-qPCR nor in microarray analysis. We chose a concentration of 100 nM tacrolimus for all subsequent studies, as this had a significant effect on most genes tested without reaching saturation.

We also monitored the time course of mRNA expression from day 0 (time point of drug application) to day 5 in the presence of 100 nM tacrolimus. Nox4, transgelin, and procollagen α1(V) mRNA expression levels were significantly elevated after one day (Nox4 and transgelin), or two days (procollagen α1(V)), resp. (Fig. 1). Although tacrolimus had no effect on α-SMA mRNA expression, we observed a constant increase from day 1 to day 5 in both tacrolimus-treated and untreated cells. This was most likely a reaction to the serum deprivation during the treatment period.

### Tacrolimus activates the TGF-β/Smad pathway, but in a ligand-independent way

**Figure 3:**
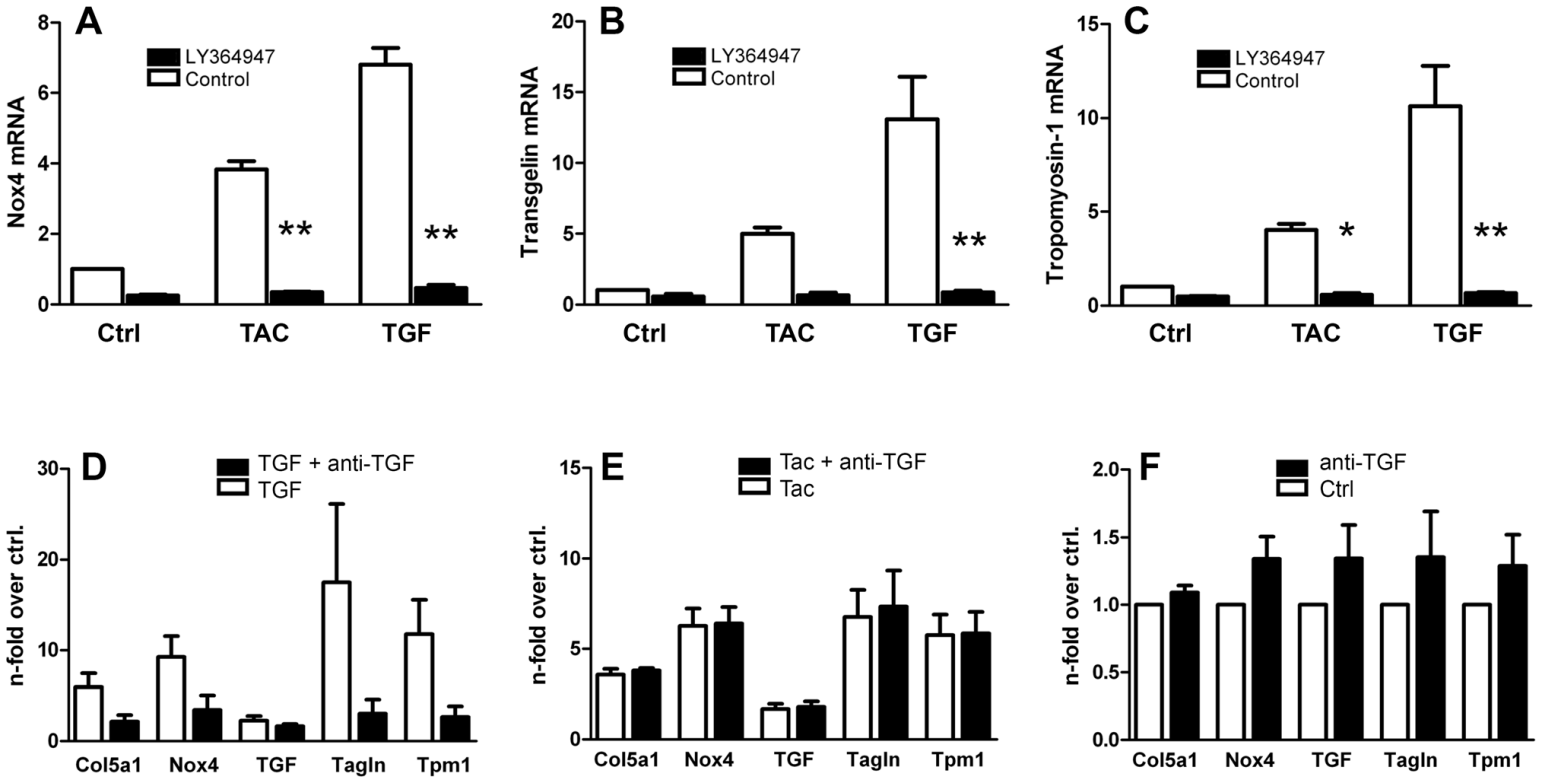
Effects of TGF-β1 signaling blockade. TK-173 cells exposed to 100 nM tacrolimus (TAC), or 10 ng/ml TGF-β1 (TGF) for three days showed increased expression of Nox4 (A), transgelin (B), and tropomyosin-1 (C). Blockade of TGF signaling by TGF-β1 RI inhibitor LY364947 inhibited both the reactions to TGF-β1 and tacrolimus [p≤0,05 (*), and p≤0,001 (**), resp.]. In contrast, application of anti-TGF-β antibody to the medium left the reaction to tacrolimus unaffected (E), but prevented the effects of 5 ng/ml TGF-β1 on the expression of procollagen α1(V) (Col5a1), Nox4, transgelin (Tagln), and tropomyosin-1 (Tmp1) almost completely (D). Antibodies had no effect in unstimulated control cultures (F). All values were normalized to untreated control cultures.

As several fibrosis-related genes were positively regulated by tacrolimus, we hypothesized involvement of the TGF-β/Smad signaling pathway. When we treated TK-173 fibroblasts with the pro-fibrotic cytokine TGF-β1 (10 ng/ml), expression of Nox4, transgelin, and tropomyosin 1 was induced as in tacrolimus-treated cells, but with higher amplitudes (Fig. 3, A–C). Blocking TGF-β signaling with the TGF-β type I receptor kinase inhibitors LY364947 (3 µM) omitted both the reaction to tacrolimus and TGF-β1, and suppressed gene expression below control levels, suggesting that tacrolimus might act through activation of the TGF-β receptor.

**Figure 4:**
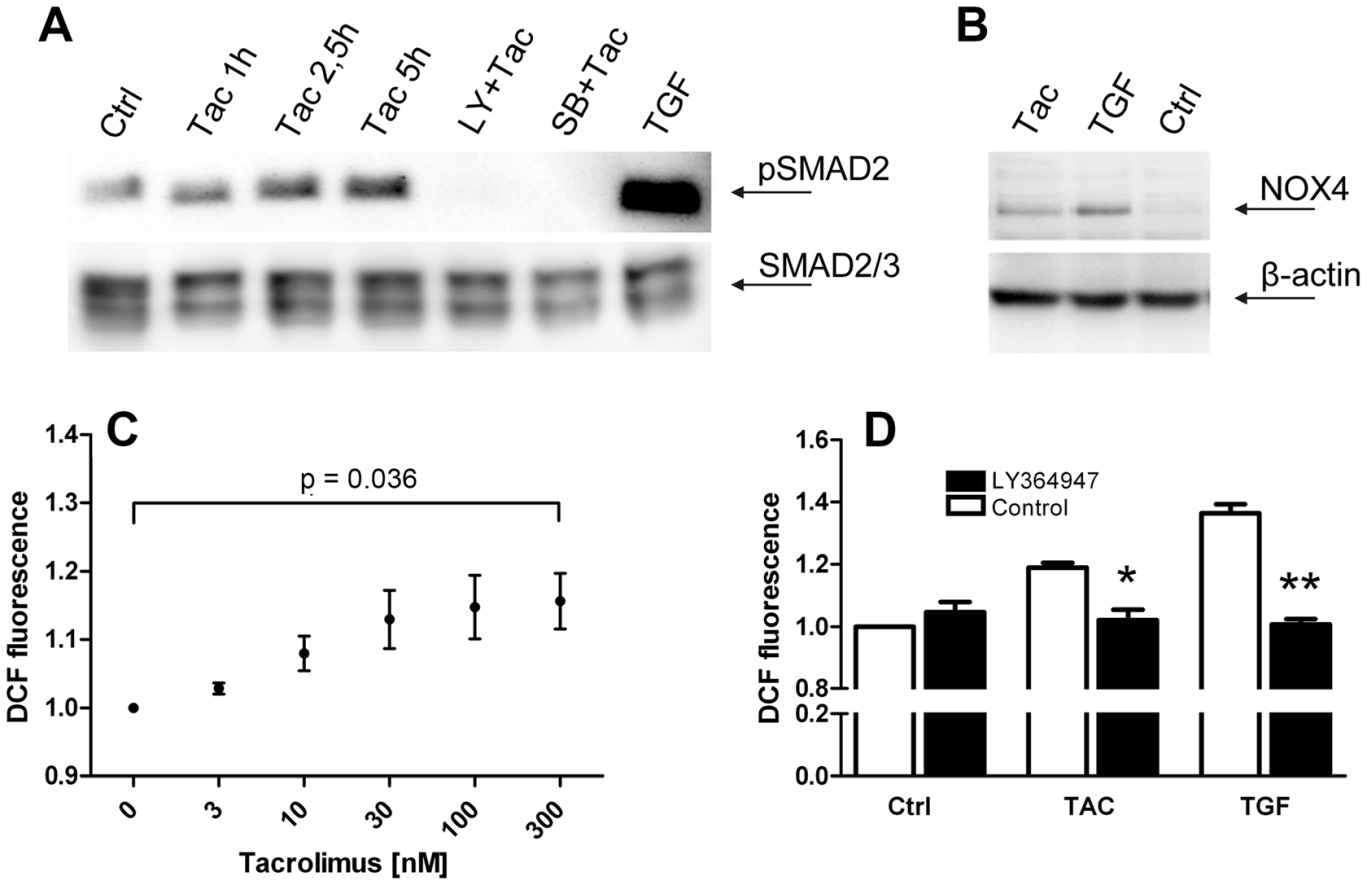
SMAD2 phosphorylation, and NOX4 expression and activity. Basal SMAD2 phosphorylation in untreated cells (Ctrl) is increased after 1 h, 2,5 h, and 5 h exposure to 100 nM tacrolimus (Tac). Preincubation with the TGF-β1-RI kinase inhibitors LY364947, or SB-431542 before exposure to tacrolimus (LY+Tac, and SB+Tac, resp.) ablates phosphorylation of SMAD2. 10 ng/ml TGF-β1 (TGF) served as a positive control. pSMAD2 band appeared at approx. 55 kD (A). Western blot for NOX4 protein in TK-173 fibroblasts treated over three days with 100 nM tacrolimus (Tac), 10 ng/ml TGF-β1 (TGF), and untreated control cells (Ctrl). A specific Nox4 band at 60 kD is present after stimulation of the cells with tacrolimus or TGF-β1, but undetectable in control cells (B). Intracellular H_2_O_2_ levels in TK-173 cells, expressed as relative DCF (dichlorofluorescin) fluorescence, were increased by tacrolimus in a concentration-dependent manner, following a sigmoid curve (C). Co-application of the TGF-β1-RI kinase inhibitor LY364947 significantly reduced intracellular H_2_O_2_ concentrations to control levels in cells treated with 100 nM tacrolimus (TAC), or 10 ng/ml TGF-β1 (D). Results were normalized to untreated control cells.

Western blotting revealed basal SMAD2 phosphorylation in untreated controls, and increasing pSMAD2 levels after 1,5 h, 2,5 h, and 5 h of exposure to tacrolimus (Fig. 4A). Incubation of the cells with the TGF-β receptor blockers LY364947 (3 µM), or SB-431542 (10 µM) for 30 min before tacrolimus application completely inhibited SMAD2 phosphorylation.

As we had also measured a slight increase in TGF-β1 mRNA in tacrolimus-treated TK-173 fibroblasts, we utilized antibodies against TGF-β to block possible autocrine TGF-β receptor activation. A concentration of 300 ng/ml of anti-TGF-β antibody in the medium was sufficient to almost completely suppress the effects of 5 ng/ml TGF-β1 (positive control) on the expression of procollagen α1(V), Nox4, transgelin, and tropomyosin 1 (Fig. 3D). In contrast, antibody-mediated depletion of extracellular TGF-β did not change the response of fibroblasts to tacrolimus exposure (Fig. 3E). Thus, TGF-β receptor activation by tacrolimus is not dependent on presence of the specific ligand in the medium.

### Rapamycin, but not CsA has a similar action on gene expression as tacrolimus

**Figure 5:**
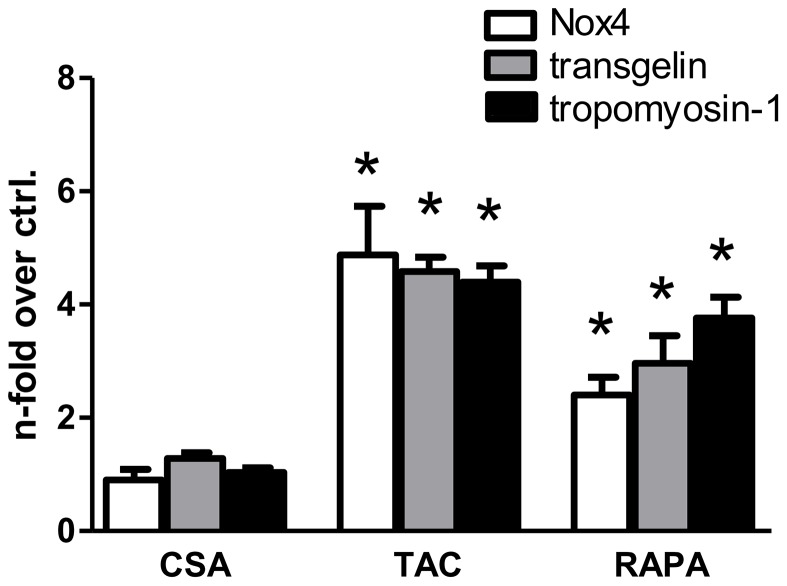
Comparison of the immunosuppressants cyclosporine A, tacrolimus, and rapamycin. Both tacrolimus (TAC; 100 nM) and rapamycin (RAPA, 100 nM) induced mRNA for Nox4, transgelin, and tropomyosin-1 after three days, although with a slightly different pattern. Cyclosporine A (CSA; 1 µM) had no effect on mRNA levels. All values normalized to untreated control. (*) denotes significance from untreated cells p≤0,05.

CsA and rapamycin (sirolimus) are the two other most widely used immunosuppressants besides tacrolimus. The use of different concentrations (1 mM of CsA, and 100 nM of tacrolimus and rapamycin, resp.) in our experiments was intended to roughly reflect the differences in actual therapeutic doses. With respect to the three genes of interest, effects of rapamycin on mRNA expression in RT-qPCR experiments were comparable to tacrolimus, with significantly positive regulation of Nox4, transgelin and tropomyosin-1 (Fig. 5). CsA had no effect on the expression of the genes tested.

### Tacrolimus-induced Nox4 protein expression is associated with increased H_2_O_2_ levels

In Western blots, Nox4 protein was below detection level in the control cultures. Tacrolimus-treated fibroblasts (100 nM, 3 days) showed a weak, but clearly positive signal for Nox4 at approx. 60 kD, while TGF-β1 treatment (10 ng/ml, 3 days) induced a stronger response (Fig. 4B).

The increased levels in Nox4 protein resulted in increased intracellular ROS levels. NAD(P)H oxidases are generally thought to produce superoxide anion (26), but our initial attempts to detect intracellular Nox4-generated superoxide anion by nitroblue tetrazolium conversion [27] failed (data not shown). However, fluorometric measurement of intracellular H_2_O_2_ by dichlorofluorescin diacetate conversion revealed increased peroxide concentrations in tacrolimus-treated (max. 1,2-fold) and TGF-β1-treated fibroblasts (max. 1,4-fold) (Fig. 4C). Concentration dependence followed a sigmoid curve, comparable to Nox4 mRNA expression curves in the RT-qPCR experiments. Treatment with the TGF-β-receptor 1 kinase activity blocker LY364947 prevented the effects of both tacrolimus and TGF-β1 and completely abated the increased H_2_O_2_ levels (Fig. 4D).

### Nox4 expression levels regulate expression of procollagen α1(V)

**Figure 6:**
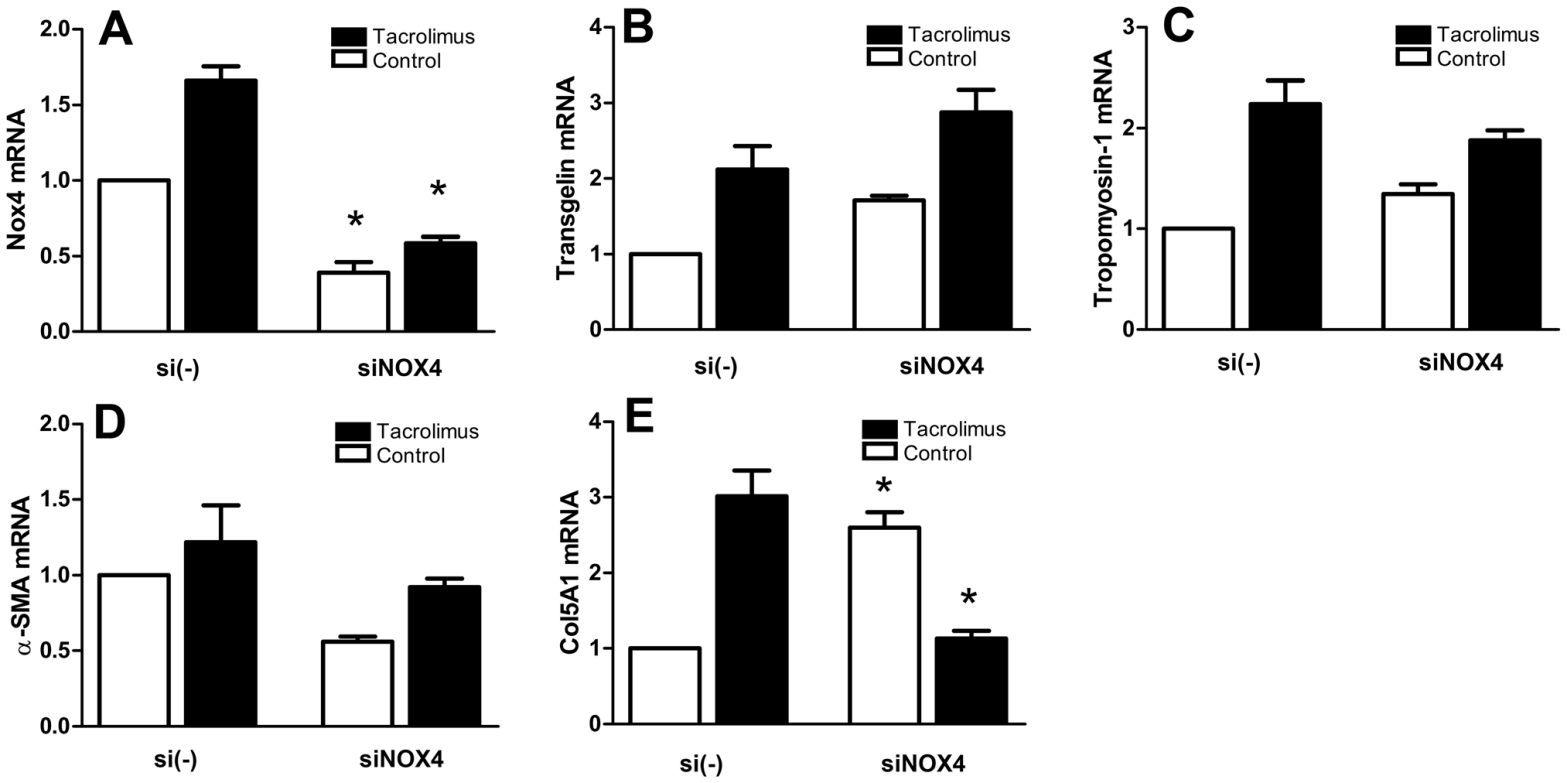
Effect of siRNA-mediated Nox-4 knock-down in human TK-173 fibroblasts. Cells were transfected with Nox4-targeted siRNA (siNOX4), or non-target siRNA (si(-)) as a control, resp., and exposed to 100 nM tacrolimus, or control conditions, for three days. Transfection with Nox4-targeted siRNA reduced Nox4 mRNA expression by 61% (untreated control), and 64% (100 nM tacrolimus), resp., compared to non-target siRNA transfected cells (A). Nox4 knock-down did not have significant effects on transgelin (B), tropomyosin-1 (C), and alpha-smooth muscle actin (D) mRNA expression, but induced a significant up-regulation of mRNA for procollagen α1(V) in untreated cells, and a down-regulation to control levels in tacrolimus-treated cells (E). Results were normalized to control-transfected, untreated cells. (*) denotes significant (p≤0,05) differences between control-transfected and siRNA-transfected cells.

There are as of yet no known specific inhibitors of Nox4, therefore we used siRNA-mediated gene knock-down to suppress Nox4 activity. Transfection of the cells with Nox4-targeted siRNA decreased Nox4 mRNA expression significantly by approx. two thirds compared to scrambled-RNA transfected cells, both in tacrolimus-treated cells and untreated cells (Fig. 6A). Nox4 knock-down had the most intense effect on procollagen α1(V), where mRNA expression was increased 2,6-fold in the control cells, and suppressed to control levels in the tacrolimus-treated cells (both compared to scrambled-RNA transfected cells) (Fig. 6E). Nox4 knock-down had no significant effects on the expression of cytoskeletal proteins (α-SMA, transgelin, and tropomyosin-1) both in tacrolimus-stimulated and control cells.

## Discussion

Induction of ligand-independent (“aberrant”) TGF-β signaling by tacrolimus has been reported in literature [28], [29]. The underlying mechanism was first described by Chen *et al.* in 1997 [30]: The FK506-binding protein 12 (FKBP12) binds to TGF-β-receptor subunit I (TGF-β-R1) [31] and anticipates the spontaneous phosphorylation of Smad 2 and 3 in the absence of ligand. As tacrolimus (FK506) occupies the TGF-β-R1-binding pocket of FKBP12 (both share the same binding region on FKBP12), interaction of FKBP12 with the receptor is inhibited and its negative modulatory effect on TGF-β signaling is lost. This results in a “leaky” receptor, which constantly activates the Smad 2/3 signaling cascade by phosphorylation, even in the complete absence of ligand. The saturation of the tacrolimus-induced TGF-β signaling at >300 nM in our experiments could indicate the complete depletion of free FKBP12 and therefore maximum aberrant signaling of the TGF-β receptor. As 10 ng/ml TGF-β1 had a markedly stronger effect on gene expression than tacrolimus, it is evident that TGF-β receptor signaling cannot be fully activated by tacrolimus-mediated FKBP12 depletion alone. The similar response of the cells to rapamycin further supports the concept above as rapamycin binds to FKBP12 at the same macrolide binding site as tacrolimus [32]. In contrast, cyclosporine-treated TK-173 cells did not show any signs of TGF-β receptor activation. Pro-fibrotic effects of cyclosporine and tacrolimus are obviously, at least in part, triggered by different pathways, although both drugs act as calcineurin inhibitors.

In untreated fibroblasts, the TGF-β receptor I blocker LY364947, but not TGF-β neutralizing antibodies, attenuated the expression of transgelin and tropomyosin-1 well below control levels (Figs. 3B, 3C). This findings, together with basal SMAD2 phosphorylation in Western blots from control cells, indicate a constant level of aberrant TGF-β signaling in untreated TK-173 fibroblasts.

The pro-fibrotic characteristics of TGF-β are well accepted [33], and a constantly increased Smad 2/3 signaling in fibroblasts by aberrant TGF-β receptor activation might at least partly contribute to kidney fibrosis under tacrolimus regimen. TGF-β induces an “activated state” in fibroblasts, the so-called myofibroblast, which is characterized by a partially smooth-muscle-like cell type with contractile properties and increased matrix deposition. The differentiation into a smooth-muscle-like cell type with contractile properties requires reorganization of the cytoskeleton, and the cytoskeletal components smooth-muscle actin, transgelin, and tropomyosin are well defined markers for this transformation [34], [35]. The actin-associated protein transgelin is one of the earliest markers in smooth muscle differentiation [36].

The renal fibroblast cell line TK-188, which was isolated from fibrotic kidney by the same group as the non-fibrotic TK-173 cells, had been shown to have a five-fold higher basal expression of α-SMA mRNA than TK-173 cells [37]. In our in-vitro system, we could not observe an increase in alpha smooth muscle actin (α-SMA) expression in TK173 cells, neither by tacrolimus, nor by TGF-β1 (TGF-β1 data not shown). However, cells slowly increased α-SMA mRNA expression as a reaction to serum deprivation. Taking the up-regulation of the cytoskeletal components tropomyosin and transgelin into account, the fibroblasts are likely missing an additional factor to fully differentiate into myofibroblasts under the given culture conditions. Desmouliere *et al.* proposed an intermediate “proto-myofibroblast” state induced by mechanical stress, which can then differentiate further into a fully activated fibroblast in the presence of TGF-β [38].

The increased release of extracellular matrix compounds by activated myofibroblasts contributes to histologic changes in fibrotic tissue. Interstitial fibrosis is characterized by accumulation of type I and III collagens, but no up-regulation of these collagens was observed in our microarray data. The ECM component most intensely regulated by tacrolimus in our experiments was procollagen α1(V). Although collagen V normally represents only 1–3% of collagen fibers, it plays a central role in the regulation of fibrillogenesis [39]. Increased deposition of collagen V in the interstitial ECM has been reported in patients with chronic renal disease [40]. Mozes *et al.* [41] found trace amounts of collagen V within the mesangium of kidneys from transgenic mice with TGF-β overexpression, while it was essentially absent in wild-type mice.

Nox4 has been reported to play an important role in the activation of fibroblasts, including heart, lung, and kidney [12], [14], [42]. It is thought that Nox4 activity shifts the redox potential within the cell and thereby modulates redox-sensitive pathways (e.g. MAP kinases). The strong impact of Nox4 knockdown on procollagen α1(V) expression indicates that the corresponding signaling pathways are redox-sensitive and respond to Nox4-generated H_2_O_2_-levels. The other genes tested were not impaired by Nox4 knock-down, so we assume that Nox4 is not generally mandatory for pro-fibrotic effects of tacrolimus-mediated TGF-β signaling.

While the other NAD(P)H oxidases release superoxide anion, Nox4 produces H_2_O_2_ as an intrinsic function of distinct regions in the protein's E-loop [43]. The tacrolimus-concentration dependent curve of intracellular H_2_O_2_ (Fig. 4C) mirrored the corresponding Nox4 mRNA expression curve (Fig. 2A) very well. This is consistent with data from literature, which suggest that Nox4 is constitutively active and its activity is regulated only by expression levels [13]. The DCF-DA assay only provides us with relative values for intracellular H_2_O_2_, as it is not possible to calibrate results. Taking the strong background into consideration, the increase in the ROS indicator fluorescence signal by max. 20% (tacrolimus) and 40% (TGF-β1) may in fact reflect a much stronger increase in H_2_O_2_ concentrations. Furthermore since blocking TGF-β signaling resulted in the suppression of Nox4 mRNA below control levels without a corresponding reduction in DCF fluorescence signal, we postulate that in unstimulated cells the Nox4-generated H_2_O_2_ levels are negligible.

In conclusion we demonstrate that tacrolimus induces aberrant TGF-β signaling in a human renal fibroblast cell line, leading to changes in cytoskeletal genes expression, intracellular H_2_O_2_ levels, and ECM synthesis. The effect is cell-type specific, as proximal tubule epithelial cells did not show it. TGF-β-like effects of tacrolimus and the structurally related rapamycin had been first described more than a decade ago, but the consideration of ligand-independent TGF-β signaling is still somehow under-represented in literature, and not all recent publications on pro-fibrotic effects of macrolide immunosuppressants take aberrant signaling into account. Furthermore, our findings in a cell culture model indicate a possible role of tacrolimus-induced Nox4 expression in fibrotic processes through modulation of collagen V expression. The mechanism behind is unclear, as Nox4 knock-down had opposite effects on collagen V expression in tacrolimus-treated and control cells. Further research on the involvement of Nox4 and collagen V in the onset of fibrosis could deepen our understanding of fibrotic processes in transplanted kidney.

## References

[1] Calne RY, White DJ, Thiru S, Evans DB, McMaster P, et al (1978) Cyclosporin A in patients receiving renal allografts from cadaver donors. Lancet 2: 1323–1327. 82836 10.1016/s0140-6736(78)91970-0

[2] Starzl TE, Todo S, Fung J, Demetris AJ, Venkataramman R, et al (1989) FK 506 for liver, kidney, and pancreas transplantation. Lancet 2: 1000–1004. 2478846 10.1016/s0140-6736(89)91014-3 PMC2966318

[3] Andreoni KA, Brayman KL, Guidinger MK, Sommers CM, Sung RS (2007) Kidney and pancreas transplantation in the United States, 1996–2005. Am J Transplant 7: 1359–1375. 17428285 10.1111/j.1600-6143.2006.01781.x

[4] Nankivell BJ, Borrows RJ, Fung CL, O'Connell PJ, Allen RD, et al (2003) The natural history of chronic allograft nephropathy. N Engl J Med 349: 2326–2333. 14668458 10.1056/NEJMoa020009

[5] Wolf G, Killen PD, Neilson EG (1990) Cyclosporin A stimulates transcription and procollagen secretion in tubulointerstitial fibroblasts and proximal tubular cells. J Am Soc Nephrol 1: 918–922. 2103851 10.1681/ASN.V16918

[6] Roos-van Groningen MC, Scholten EM, Lelieveld PM, Rowshani AT, Baelde HJ, et al (2006) Molecular comparison of calcineurin inhibitor-induced fibrogenic responses in protocol renal transplant biopsies. J Am Soc Nephrol 17: 881–888. 16467444 10.1681/ASN.2005080891

[7] Matl I, Viklicky O, Voska L, Lodererova A, Vitko S (2005) The effect of different immunosuppressive regimens on TGF-beta1 expression in kidney transplant patients. Transpl Int 18: 668–671. 15910291 10.1111/j.1432-2277.2005.00115.x

[8] Khanna A, Plummer M, Bromberek C, Bresnahan B, Hariharan S (2002) Expression of TGF-beta and fibrogenic genes in transplant recipients with tacrolimus and cyclosporine nephrotoxicity. Kidney Int 62: 2257–2263. 12427154 10.1046/j.1523-1755.2002.00668.x

[9] Border WA, Brees D, Noble NA (1994) Transforming growth factor-beta and extracellular matrix deposition in the kidney. Contrib Nephrol 107: 140–145. 8004960 10.1159/000422972

[10] Border WA, Noble NA (1994) Transforming growth factor beta in tissue fibrosis. N Engl J Med 331: 1286–1292. 7935686 10.1056/NEJM199411103311907

[11] Slattery C, Campbell E, McMorrow T, Ryan MP (2005) Cyclosporine A-induced renal fibrosis: a role for epithelial-mesenchymal transition. Am J Pathol 167: 395–407. 16049326 10.1016/S0002-9440(10)62984-7 PMC1603578

[12] Bondi CD, Manickam N, Lee DY, Block K, Gorin Y, et al (2010) NAD(P)H oxidase mediates TGF-beta1-induced activation of kidney myofibroblasts. J Am Soc Nephrol 21: 93–102. 19926889 10.1681/ASN.2009020146 PMC2799274

[13] Bedard K, Krause KH (2007) The NOX family of ROS-generating NADPH oxidases: physiology and pathophysiology. Physiol Rev 87: 245–313. 17237347 10.1152/physrev.00044.2005

[14] Hecker L, Vittal R, Jones T, Jagirdar R, Luckhardt TR, et al (2009) NADPH oxidase-4 mediates myofibroblast activation and fibrogenic responses to lung injury. Nat Med 15: 1077–1081. 19701206 10.1038/nm.2005 PMC2743335

[15] Geiszt M, Kopp JB, Varnai P, Leto TL (2000) Identification of renox, an NAD(P)H oxidase in kidney. Proc Natl Acad Sci U S A 97: 8010–8014. 10869423 10.1073/pnas.130135897 PMC16661

[16] Gorin Y, Ricono JM, Wagner B, Kim NH, Bhandari B, et al (2004) Angiotensin II-induced ERK1/ERK2 activation and protein synthesis are redox-dependent in glomerular mesangial cells. Biochem J 381: 231–239. 15027896 10.1042/BJ20031614 PMC1133781

[17] Shiose A, Kuroda J, Tsuruya K, Hirai M, Hirakata H, et al (2001) A novel superoxide-producing NAD(P)H oxidase in kidney. J Biol Chem 276: 1417–1423. 11032835 10.1074/jbc.M007597200

[18] Wu RF, Ma Z, Liu Z, Terada LS (2010) Nox4-derived H2O2 mediates endoplasmic reticulum signaling through local Ras activation. Mol Cell Biol 30: 3553–3568. 20457808 10.1128/MCB.01445-09 PMC2897542

[19] Goettsch C, Goettsch W, Muller G, Seebach J, Schnittler HJ, et al (2009) Nox4 overexpression activates reactive oxygen species and p38 MAPK in human endothelial cells. Biochem Biophys Res Commun 380: 355–360. 19280689 10.1016/j.bbrc.2009.01.107

[20] Mandal CC, Ganapathy S, Gorin Y, Mahadev K, Block K, et al (2011) Reactive oxygen species derived from Nox4 mediate BMP2 gene transcription and osteoblast differentiation. Biochem J 433: 393–402. 21029048 10.1042/BJ20100357 PMC4539275

[21] Amara N, Goven D, Prost F, Muloway R, Crestani B, et al (2010) NOX4/NADPH oxidase expression is increased in pulmonary fibroblasts from patients with idiopathic pulmonary fibrosis and mediates TGFbeta1-induced fibroblast differentiation into myofibroblasts. Thorax 65: 733–738. 20685750 10.1136/thx.2009.113456 PMC3004009

[22] Li J, Stouffs M, Serrander L, Banfi B, Bettiol E, et al (2006) The NADPH oxidase NOX4 drives cardiac differentiation: Role in regulating cardiac transcription factors and MAP kinase activation. Mol Biol Cell 17: 3978–3988. 16775014 10.1091/mbc.E05-06-0532 PMC1556380

[23] Borbely G, Szabadkai I, Horvath Z, Marko P, Varga Z, et al (2010) Small-molecule inhibitors of NADPH oxidase 4. J Med Chem 53: 6758–6762. 20731357 10.1021/jm1004368

[24] Schuttert JB, Liu MH, Gliem N, Fiedler GM, Zopf S, et al (2003) Human renal fibroblasts derived from normal and fibrotic kidneys show differences in increase of extracellular matrix synthesis and cell proliferation upon angiotensin II exposure. Pflugers Arch 446: 387–393. 12684791 10.1007/s00424-003-1026-y

[25] Jennings P, Koppelstaetter C, Aydin S, Abberger T, Wolf AM, et al (2007) Cyclosporine A induces senescence in renal tubular epithelial cells. Am J Physiol Renal Physiol 293: F831–838. 17596534 10.1152/ajprenal.00005.2007

[26] Wieser M, Stadler G, Jennings P, Streubel B, Pfaller W, et al (2008) hTERT alone immortalizes epithelial cells of renal proximal tubules without changing their functional characteristics. Am J Physiol Renal Physiol 295: F1365–1375. 18715936 10.1152/ajprenal.90405.2008

[27] Choi HS, Kim JW, Cha YN, Kim C (2006) A quantitative nitroblue tetrazolium assay for determining intracellular superoxide anion production in phagocytic cells. J Immunoassay Immunochem 27: 31–44. 16450867 10.1080/15321810500403722

[28] Goldfarb DA (2003) Expression of TGF-beta and fibrogenic genes in transplant recipients with tacrolimus and cyclosporine nephrotoxicity. J Urol 169: 2436–2437. 14558568

[29] Khanna AK (2003) The immunosuppressive agent tacrolimus induces p21WAF/CIP1WAF1/CIP1 via TGF-beta secretion. Biochem Biophys Res Commun 303: 266–272. 12646197 10.1016/s0006-291x(03)00340-1

[30] Chen YG, Liu F, Massague J (1997) Mechanism of TGFbeta receptor inhibition by FKBP12. EMBO J 16: 3866–3876. 9233797 10.1093/emboj/16.13.3866 PMC1170011

[31] Wang T, Donahoe PK, Zervos AS (1994) Specific interaction of type I receptors of the TGF-beta family with the immunophilin FKBP-12. Science 265: 674–676. 7518616 10.1126/science.7518616

[32] Van Duyne GD, Standaert RF, Karplus PA, Schreiber SL, Clardy J (1993) Atomic structures of the human immunophilin FKBP-12 complexes with FK506 and rapamycin. J Mol Biol 229: 105–124. 7678431 10.1006/jmbi.1993.1012

[33] Pohlers D, Brenmoehl J, Loffler I, Muller CK, Leipner C, et al (2009) TGF-beta and fibrosis in different organs - molecular pathway imprints. Biochim Biophys Acta 1792: 746–756. 19539753 10.1016/j.bbadis.2009.06.004

[34] Popova AP, Bozyk PD, Goldsmith AM, Linn MJ, Lei J, et al (2010) Autocrine production of TGF-beta1 promotes myofibroblastic differentiation of neonatal lung mesenchymal stem cells. Am J Physiol Lung Cell Mol Physiol 298: L735–743. 20190033 10.1152/ajplung.00347.2009 PMC2886615

[35] Mayer DC, Leinwand LA (1997) Sarcomeric gene expression and contractility in myofibroblasts. J Cell Biol 139: 1477–1484. 9396753 10.1083/jcb.139.6.1477 PMC2132619

[36] Assinder SJ, Stanton JA, Prasad PD (2009) Transgelin: an actin-binding protein and tumour suppressor. Int J Biochem Cell Biol 41: 482–486. 18378184 10.1016/j.biocel.2008.02.011

[37] Dihazi H, Dihazi GH, Mueller C, Lahrichi L, Asif AR, et al (2011) Proteomics characterization of cell model with renal fibrosis phenotype: osmotic stress as fibrosis triggering factor. J Proteomics 74: 304–318. 21118732 10.1016/j.jprot.2010.11.007

[38] Desmouliere A, Chaponnier C, Gabbiani G (2005) Tissue repair, contraction, and the myofibroblast. Wound Repair Regen 13: 7–12. 15659031 10.1111/j.1067-1927.2005.130102.x

[39] Wenstrup RJ, Florer JB, Brunskill EW, Bell SM, Chervoneva I, et al (2004) Type V collagen controls the initiation of collagen fibril assembly. J Biol Chem 279: 53331–53337. 15383546 10.1074/jbc.M409622200

[40] Vleming LJ, Baelde JJ, Westendorp RG, Daha MR, van Es LA, et al (1995) Progression of chronic renal disease in humans is associated with the deposition of basement membrane components and decorin in the interstitial extracellular matrix. Clin Nephrol 44: 211–219. 8575119

[41] Mozes MM, Bottinger EP, Jacot TA, Kopp JB (1999) Renal expression of fibrotic matrix proteins and of transforming growth factor-beta (TGF-beta) isoforms in TGF-beta transgenic mice. J Am Soc Nephrol 10: 271–280. 10215326 10.1681/ASN.V102271

[42] Cucoranu I, Clempus R, Dikalova A, Phelan PJ, Ariyan S, et al (2005) NAD(P)H oxidase 4 mediates transforming growth factor-beta1-induced differentiation of cardiac fibroblasts into myofibroblasts. Circ Res 97: 900–907. 16179589 10.1161/01.RES.0000187457.24338.3D

[43] Takac I, Schroder K, Zhang L, Lardy B, Anilkumar N, et al (2011) The E-loop is involved in hydrogen peroxide formation by the NADPH oxidase Nox4. J Biol Chem 286: 13304–13313. 21343298 10.1074/jbc.M110.192138 PMC3075677

